# The association between remnant cholesterol and the risk of osteoporotic vertebral fracture in older adults

**DOI:** 10.1371/journal.pone.0327171

**Published:** 2025-07-29

**Authors:** Changzhi Liu, He Tong, Xifa Gao, Jiangchuan Wang, Zicheng Wei, Yu Wang, Jianhua Wang, Xiao Chen

**Affiliations:** 1 Department of Radiology, Funan County People’s Hospital, Funan Hospital Affiliated with the Medical College of Fuyang Normal University, Fuyang, China; 2 Department of Radiology, Second Affiliated Hospital of Bengbu Medical University, Bengbu, China; 3 Department of Radiology, Affiliated Hospital of Nanjing University of Chinese Medicine, Nanjing, China; Shanghai Jiaotong University: Shanghai Jiao Tong University, CHINA

## Abstract

**Background:**

Serum lipid levels have been shown to influence bone mineral density. Additionally, a limited number of studies have suggested that remnant cholesterol (RC) may be linked to the risk of osteoporosis. However, the relationship between RC and fracture risk remains unclear. This study aimed to explore the association between RC levels and the risk of vertebral fractures in a longitudinal cohort.

**Methods:**

A total of 1995 participants aged 50 years or older who underwent chest computed tomography (CT) scans for lung cancer screening between July 2016 and December 2019 were included in this study. Follow-up continued until June 2023. The concentration of RC was calculated via the following formula: total cholesterol minus the sum of high-density lipoprotein cholesterol and low-density lipoprotein cholesterol. The RC-to-cholesterol ratio was also determined. The participants were divided into low and high groups for RC, and the RC-to-cholesterol ratio was based on the median values. Vertebral fractures were assessed via the Genant semiquantitative classification system on CT-reconstructed sagittal images.

**Results:**

During a median follow-up period of 60 months, 95 new vertebral fractures were recorded. The incidence of fractures was significantly greater among participants with low RC levels than among those with high RC levels (6.4% vs. 3.1%, P < 0.01). A multivariate Cox proportional hazards model indicated that individuals with high RC levels had a 41% lower risk of vertebral fractures than those with low RC levels did (adjusted hazard ratio [aHR]: 0.48, 95% confidence interval [CI]: 0.24--0.93). Similar findings were observed for the RC-to-cholesterol ratio (aHR: 0.40, 95% CI: 0.21–0.79). Restricted cubic spline analysis further demonstrated that the risk of vertebral fractures decreased as the RC level and the RC-to-cholesterol ratio increased. Subgroup analysis revealed that the association between RC and fracture risk was mainly observed in women.

**Conclusion:**

Higher levels of remnant cholesterol and a higher RC-to-cholesterol ratio were associated with a reduced risk of vertebral fractures, particularly in women.

## Introduction

Osteoporosis is a skeletal disorder characterized by compromised bone strength, which predisposes individuals to an increased risk of fractures. Aging, female sex, menopause and prolonged use of corticosteroids are the main risk factors for osteoporosis [1]. Dyslipidemia, on the other hand, refers to abnormal levels of lipids in the blood. Although traditionally considered separately, recent studies have indicated that lipid metabolism may influence bone health. Oxidized lipids and cholesterol can affect the differentiation of mesenchymal stem cells (MSCs) and stimulate osteoblasts to secrete receptor activator of nuclear factor-kappa B ligand (RANKL), inducing excessive bone resorption [2]. Lipid imbalances are also related to the secretion of inflammatory factors, such as interleukin-1 (IL-1), IL-6 and tumor necrosis factor-α (TNF-α), which are associated with osteoclast formation and bone resorption [3]. Triglycerides, total cholesterol (TC), and low-density lipoprotein cholesterol (LDL-c) are negatively associated with whole-body bone mineral density (BMD) [4–6]. Conversely, higher levels of high-density lipoprotein cholesterol (HDL-c) may be associated with increased BMD and a reduced risk of osteoporosis [7]. A recent Mendelian randomization study also revealed a link between dyslipidemia and osteoporosis [8]. However, the associations between serum lipids and BMD or osteoporosis have not been confirmed because the findings are not always consistent [9,10].

Fracture is the most severe clinical outcome of osteoporosis. Interestingly, some studies have also shown an association between serum lipid levels and fracture risk. Sivas et al. [11] demonstrated that an increase in TC was associated with a decreased risk of vertebral fracture. However, a meta-analysis indicated that the serum TC concentration was positively associated with the risk of bone fracture [12]. U-shaped relationships were observed between HDL-c and LDL-c and hip fracture risk [13]. In contrast, a recent study revealed an association between HDL-c levels and fracture risk in age groups ≥ 60 years but not in age groups < 60 years [14]. Similar results were reported in healthy older adults > 65 years [15]. Given this information, the association between serum lipids and fracture risk has also not reached a conclusion because the findings are not always consistent [16].

Remnant cholesterol (RC) refers to the cholesterol components in the blood that are in addition to LDL-c and HDL-c. RC primarily originates from dietary cholesterol and lipoproteins synthesized by the liver, including lipoprotein remnants such as very-low-density lipoprotein (VLDL) remnants and chylomicron remnants. An increasing number of studies have indicated that an elevated level of RC is associated with an increased risk of various cardiovascular diseases [16–18]. Recent studies have shown that RC is also associated with the risk of low bone mass [9,19], which indicates that RC may be a novel factor associated with bone metabolism. Fracture risk is the most important clinical outcome of osteoporosis and is a more clinically relevant issue. However, to our knowledge, few studies have shown an association between RC and osteoporotic vertebral fracture [20]. In this study, the association between RC and the risk of osteoporotic vertebral fracture was explored in a Chinese population.

## Methods

### Study design and participants

This retrospective longitudinal study was performed at the Affiliated Hospital of Nanjing University of Chinese Medicine between July 2016 and December 2019. The study population has been previously described [21]. In summary, a total of 1995 participants aged 50 years or older who underwent chest computed tomography (CT) scans for lung cancer screening were included in this study.

Participants were excluded if they had only one CT scan, had vertebral fractures at baseline, had traumatic fractures, had vertebral metastases or tumors, had metabolic bone diseases, or had received glucocorticoid therapy for more than three months. We collected all the data from the electronic medical records. The participants’ medical history information in their medical records was obtained through self-reports. The baseline data were obtained mainly from October 24, 2019, to October 23, 2020. The authors could not access information that could identify individual participants during or after data collection. Ethics approval was obtained from the Affiliated Hospital of Nanjing University of Chinese Medicine. The study was conducted in accordance with the Declaration of Helsinki. The need for informed consent was waived by the Ethics Committee of the affiliated hospital of Nanjing University of Chinese Medicine because of the retrospective nature of the study.

### Data collection

Demographic data, such as age and sex, and laboratory test data, such as liver and renal function markers, blood glucose, serum albumin and serum lipid biochemical indicators (HDL-c; LDL-c; TC; triglyceride (TG)) were collected from medical records. Serum lipids were determined by an automatic biochemical analyzer (Hitachi 7180, Japan) while the samples were in a fasting state. All tests were performed at our institution via the same methods. Two people performed the data collection (XG and JW). The RC concentration was calculated via the following equation: TC – (HDL-c + LDL-c). The ratio of RC to cholesterol was also calculated.

### Bone mass and fracture assessment

In our study, we did not collect bone mineral density (BMD) data for each participant. However, bone CT values have been shown to be strongly correlated with potential BMD [22]. Therefore, we used bone CT attenuation as a proxy to assess bone mass. The CT scan parameters and bone CT attenuation measurements (expressed in Hounsfield units, HU) have been previously described [21]. In brief, the CT scans were performed with settings of 120 kV, 100–120 mAs, and a slice thickness of 0.625 mm. To measure bone CT attenuation, we placed a 2–3 cm region of interest (ROI) over the trabecular bone at the mid-level of the T11 vertebral body on axial CT images. Vertebral fractures were assessed via reconstructed CT sagittal images and classified according to the Genant semiquantitative method [23]. A fracture was defined as a reduction in vertebral height by more than 20%. The occurrence of fractures was assessed every year. The follow-up period concluded in June 2023. If a new fracture occurred, follow-up was terminated at the time of fracture detection. The spine from the T1 vertebra to the L2 vertebra was assessed.

### Ethical statements

This study was approved by the Ethics Committee of the Affiliated Hospital of Nanjing University of Chinese Medicine (2019-NL-15) and was conducted in accordance with the Declaration of Helsinki. Informed consent was waived because this was a retrospective study.

### Statistical analysis

We used SPSS software (SPSS 25.0) and R software (R 4.3.0.) for statistical analysis. The quantitative data are presented as the means (standard deviations), and the categorical data are presented as numbers (percentages). The participants were divided into low and high groups on the basis of the median RC level and the RC/cholesterol ratio. The normality of the data was assessed via the Kolmogorov‒Smirnov test. Data analysis was performed via independent two-sample t tests (data with a normal distribution) or Mann‒Whitney U tests (data with an abnormal distribution) for quantitative data. Categorical data were analyzed via chi-square tests or Fisher’s exact test. The associations between fracture risk and remnant cholesterol levels were analyzed via a multivariate Cox proportional hazards model, Kaplan‒Meier analysis and restricted cubic splines. RC was divided into two groups on the basis of the median (3.2 mmol/L). The following variables were adjusted for: age; sex; bone CT value; diabetes mellitus status; and serum ALB, HDL-c, TC, and TG levels. Restricted cubic splines were assessed via R software (R 4.3.0.) were also used to determine the associations between the risk of vertebral fracture and remnant cholesterol and the remnant cholesterol/cholesterol ratio. The sample size was estimated via PASS software, with β = 0.90 and ɑ = 0.05. A hazard ratio (HR) of 2 was used, and the estimated sample size was 961 at a power of 0.9. A P value less than 0.05 was considered statistically significant.

## Results

### Characteristics of the subjects

We identified 95 cases of new vertebral fracture during a median follow-up of 50 (19–61) months. A total of 1158 men and 837 women were included. The mean age was 62.39 ± 9.53 years (Table 1). The mean HDL-c, LDL-c, TC and TG levels were 1.50, 2.95, 4.79 and 1.54 mmol/L, respectively, and the median RC was 0.32 mmol/L. A total of 1995 participants were divided into two groups on the basis of the median RC level (Table 1). The low RC group (≤ 0.32 mmol/L) had a greater average age than the high RC group did (63.33 ± 9.93 years vs 61.42 ± 9.00 years, P < 0.01). Significant differences were also observed in sex distribution and bone CT attenuation between participants with high and low RC levels (P = 0.019). The low RC group presented lower levels of serum ALB, alkaline phosphatase, total cholesterol (TC) and total triglyceride (TG) (P < 0.01 for all). The HDL-c levels were higher in the low RC group than in the high RC group.

**Table 1:** Baseline characteristics of the subjects.

	Overall	Low RC (≤ 0.32) (N = 1013)	High RC (> 0.32) (N = 982)	p
Age (years)	62.39 ± 9.53	63.33 ± 9.93	61.42 ± 9.00	< 0.01
Sex (male/female)	1158/837	608/405	550/432	0.019
Body mass index (kg/m^2^)	25.76 ± 3.12	26.35 ± 3.16	25.15 ± 2.93	< 0.01
Bone CT attenuation (HU)	141.85 ± 43.04	139.63 ± 43.09	144.14 ± 42.88	0.019
AST (U/L)	24.03 ± 10.74	23.94 ± 6.98	24.14 ± 7.57	0.66
Albumin (mmol/L)	40.76 ± 3.11	40.15 ± 3.12	41.41 ± 2.99	< 0.01
ALP (mmol/L)	77.97 ± 21.94	76.17 ± 22.98	79.83 ± 20.67	< 0.01
Creatinine (mmol/L)	77.69 ± 23.75	78.04 ± 28.50	77.34 ± 17.54	0.51
Blood glucose (mmol/L)	5.55 ± 1.40	5.55 ± 1.46	5.55 ± 1.33	0.99
HDL cholesterol (mmol/L)	1.50 ± 0.35	1.54 ± 0.35	1.46 ± 0.35	< 0.01
LDL-cholesterol (mmol/L)	2.95 ± 0.82	2.93 ± 0.86	2.98 ± 0.78	0.23
TC (mmol/L)	4.79 ± 1.02	4.43 ± 0.91	5.15 ± 1.00	< 0.01
TG (mmol/L)	1.54 ± 1.12	1.26 ± 0.64	1.82 ± 1.40	< 0.01
Diabetes	164 (8.22%)	87 (8.59%)	77 (7.84%)	0.54
Follow time (median, months)	60 (50-61)	60 (49-61)	60 (50-61)	0.05
Fracture	95 (4.76%)	65 (6.41%)	30 (3.05%)	< 0.01

AST: aspartate aminotransferase; ALP: alkaline phosphatase; CT: computed tomography; HDL-c: high-density lipoprotein cholesterol; HU: Hounsfield unit; LDL-c: low-density lipoprotein cholesterol; TC: total cholesterol; TG: triglyceride.

### Incidence of vertebral fracture

**Fig 1:**
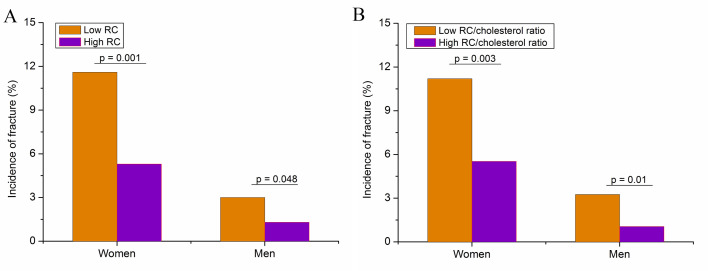
Incidence of vertebral fracture in women and men during follow-up divided by the remnant cholesterol level (A) and the remnant cholesterol/cholesterol ratio (B).

The incidence of vertebral fracture in the high RC group was significantly lower than that in the low RC group (6.41% vs 3.05%, P < 0.01) (Table 1) in the overall population. Fig. 1 shows the incidence of vertebral fracture in women and men during the follow-up divided by the RC level (Fig. 1A) and the RC/cholesterol ratio (Fig. 1B). The incidence of vertebral fracture was greater in the low RC group than in the high RC group for both women (P < 0.01) and men (P < 0.05). Similar results were observed between the low and high RC/cholesterol ratio groups (≤ 0.07 vs > 0.07).

### The association between remnant cholesterol and the risk of fracture

**Fig 2:**
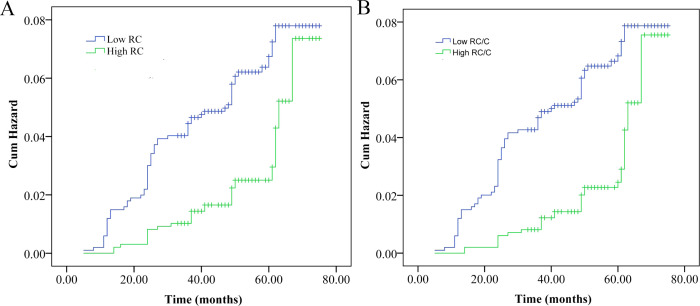
Cumulative probability curves of vertebral fracture risk divided by low and high levels of remnant cholesterol (A) or the remnant cholesterol/cholesterol ratio (B).

**Fig 3:**
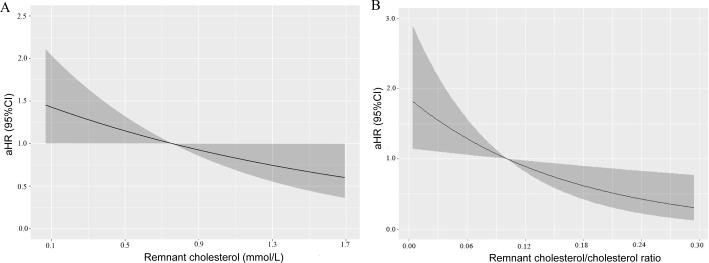
Restricted cubic splines show the multivariable adjusted hazard ratio for the risk of vertebral fracture according to the levels of remnant cholesterol (A) and the remnant cholesterol/cholesterol ratio (B). Age, sex, bone attenuation, diabetes status, body mass index, serum albumin, high-density lipoprotein cholesterol, total cholesterol, and high-density lipoprotein cholesterol were adjusted.

We subsequently explored the associations between RC and vertebral fracture via Kaplan‒Meier analysis, the Cox proportional hazards model, and restricted cubic splines. The cumulative hazard curve revealed a significantly greater risk of fracture in the low RC group than in the high RC group (log rank P < 0.01) (Fig 2A). Three adjusted models were adopted for Cox proportional hazards model analysis. All three models demonstrated a negative association between high RC levels and the risk of fracture (Table 2). The fully adjusted model showed an adjusted hazard ratio (aHR) of 0.48 (95% confidence interval (CI): 0.24–0.93). Restricted cubic splines further revealed that the risk of vertebral fracture decreased with increasing RC after adjusting for age, sex, and bone CT attenuation (Fig 3A).

**Table 2:** Association between remnant cholesterol and the risk of fracture.

	Model 1	p	Model 2	p	Model 3	p
aHR (95%CI)		aHR (95%CI)		aHR (95%CI)	
Age (years)	1.05 (1.02-1.08)	< 0.001	1.05 (1.02-1.08)	0.001	1.05 (1.02-1.08)	0.001
Sex (F vs M)	2.50 (1.55-4.03)	<0.001	2.45 (1.50-4.00)	< 0.001	2.50 (1.51-4.16)	0.001
Bone attenuation (HU)	0.97 (0.96-0.98)	< 0.001	0.97 (0.96-0.98)	< 0.001	0.97 (0.96-0.98)	< 0.001
Remnant cholesterol						
Low (< 0.32)	1		1		1	
High (> 0.32)	0.56 (0.36-0.86)	0.025	0.55 (0.34-0.88)	0.013	0.48 (0.24-0.93)	0.031

**Notes:** Model 1 included age, sex, bone attenuation and remnant cholesterol. Model 2 was further adjusted for diabetes, body mass index and serum albumin; Model 3 was additionally adjusted for high-density lipoprotein cholesterol, total cholesterol, and low-density lipoprotein cholesterol.

**Abbreviations:** aHR: adjusted hazard ratio; CI, confidence interval.

### Associations between the RC/cholesterol ratio and the risk of fracture

We also investigated the association between the RC/cholesterol ratio and the risk of fracture (Fig 2B, Table 3, Fig 3B). The cumulative hazard curve revealed a significantly greater risk of fracture in the low RC/cholesterol ratio group than in the high RC/cholesterol ratio group (log rank P < 0.01) (Fig 2B). Three adjusted models were adopted for analysis. All three Cox proportional hazards models demonstrated a negative association between high RC levels and the risk of fracture (Table 3). The fully adjusted model showed an adjusted hazard ratio (aHR) of 0.40 (95% CI: 0.21–0.79). Restricted cubic splines further revealed that the risk of vertebral fracture decreased with increasing RC/cholesterol ratios after adjustment for age, sex, and bone CT attenuation (Fig 3B).

**Table 3:** Association between the remnant cholesterol/cholesterol ratio and the risk of fracture.

	Model 1	p	Model 2	p	Model 3	p
	aHR (95%CI)		aHR (95%CI)		aHR (95%CI)	
Age (years)	1.05 (1.02-1.08)	0.001	1.05 (1.02-1.08)	0.001	1.05 (1.02-1.08)	0.001
Sex (F vs M)	2.46 (1.53-3.95)	< 0.001	2.38 (1.46-3.90)	< 0.001	2.46 (1.49-4.09)	< 0.001
Bone attenuation (HU)	0.97 (0.96-0.98)	< 0.001	0.97 (0.96-0.98)	< 0.001	0.97 (0.96-0.98)	< 0.001
Remnant cholesterol/cholesterol ratio						
Low (< 0.07)	1		1		1	
High (≥ 0.07)	0.52 (0.33-0.80)	0.003	0.50 (0.31-0.81)	0.005	0.40 (0.21-0.79)	0.008

**Notes:** Model 1 included age, sex, bone attenuation and remnant cholesterol. Model 2 was further adjusted for diabetes, body mass index and serum albumin; Model 3 was additionally adjusted for high-density lipoprotein cholesterol, total cholesterol, and low-density lipoprotein cholesterol.

**Abbreviations:** CI, confidence interval; aHR: adjusted hazard ratio.

### Subgroup analysis

A subgroup analysis divided by sex is shown in Table 4. All three Cox proportional hazards models demonstrated a negative association between high RC values or high RC/cholesterol ratios and the risk of fracture in women. The fully adjusted model yielded adjusted hazard ratios (aHRs) of 0.29 (95% CI: 0.13–0.66) and 0.29 (95% CI: 0.13–0.65). However, no such associations were observed in men.

**Table 4:** Association between the remnant cholesterol or the remnant cholesterol/cholesterol ratio and the risk of fracture divided by sex.

		Model 1	p	Model 2	p	Model 3	p
		aHR (95%CI)		aHR (95%CI)		aHR (95%CI)	
Women	Remnant cholesterol						
	Low (< 0.32)	1		1			
	High (> 0.32)	0.54 (0.32-0.89)	0.016	0.49 (0.28-0.85)	0.011	0.29 (0.13-0.66)	0.003
	Remnant cholesterol/cholesterol ratio						
	Low (< 0.07)	1		1		1	
	High (≥ 0.07)	0.54 (0.33-0.90)	0.018	0.49 (0.29-0.86)	0.012	0.29 (0.13-0.65)	0.003
Men	Remnant cholesterol						
	Low (< 0.32)	1	1	1	1	1	
	High (> 0.32)	0.23 (0.24-1.41)	0.23	0.68 (0.27-1.68)	0.40	2.04 (0.50-8.27)	0.32
	Remnant cholesterol/cholesterol ratio						
	Low (< 0.07)	1		1		1	
	High (≥ 0.07)	0.41 (0.16-1.02)	0.06	0.47 (0.18-1.23)	0.13	0.86 (0.22-3.40)	0.84

**Notes:** Model 1 was adjusted for age and bone attenuation. Model 2 was further adjusted for diabetes, body mass index and serum albumin; Model 3 was additionally adjusted for high-density lipoprotein cholesterol, total cholesterol, and low-density lipoprotein cholesterol.

**Abbreviations:** CI, confidence interval; aHR: adjusted hazard ratio.

## Discussion

Associations between serum lipid levels and BMD, osteoporosis or fracture risk have been reported. However, the results are conflicting. Moreover, the association between RC and fracture risk has not been well studied. This longitudinal study is the first to report that RC levels are associated with the risk of vertebral fracture in a Chinese population. The risk of vertebral fracture risk was 41% lower in subjects with high RCs (≥ 0.32 mmol/L) than in those with low RCs (< 0.32 mmol/L). A similar decrease (46%) was observed between subjects with a high RC/cholesterol ratio (> 0.07) and those with a low RC/cholesterol ratio (≤ 0.07). This study reported novel risk factors for vertebral fracture, which may be useful for detecting high fracture risk early, especially for women.

RC is associated with an increased risk and mortality of various cardiovascular diseases [16–18,24]. Some studies have shown that serum lipid levels may be associated with fracture risk [11–13]. However, to our knowledge, the association between RC and bone fracture is still unknown. Interestingly, two recent studies demonstrated that RC was associated with low BMD (9,19). Negative correlations between the serum RC and total spine BMD and hip BMD were observed. In other words, a high RC indicates a low BMD. These results suggest that a high level of RC may be related to a high risk of fracture. However, in the present study, we found that a high level of RC was a protective factor against fracture. The reasons for this apparent discrepancy are not clear. One of the reasons was the difference in age between our study and the two previously published studies. The mean age was greater than 60 years in the present study, which was obviously greater than those reported in published studies, which were 40 years old and younger than 60 years. The associations between RC and bone CT attenuation were studied in this study. Only a slight difference (< 5 HU) was observed between low and high RCs. In addition, the results of this study demonstrated that RC was independently associated with fracture risk even when controlling for bone CT attenuation. Therefore, it can be speculated that RC may affect fractures through pathways independent of bone density. Interestingly, a recent study revealed an association between cumulative RC and fragility fractures [20]. The results also revealed that the risk of fracture was greater in the Q1 quartile than in the Q2 quartile (HR = 1.53, 95% CI: 1.16–2.01). A greater risk was also observed in the Q4 quartile than in the Q2 quartile. This study partially showed that cumulative RC may be related to a low fracture risk to some degree. However, it may not be proper to directly compare the two studies because different references and RCs were adopted during the analyses.

Some studies have shown that obesity is related to a high risk of vertebral fracture [25,26]. Recent evidence highlights a paradoxical relationship between obesity and fracture risk in postmenopausal women. A meta-analysis demonstrated that obesity in this population is associated with elevated risks of both all-cause and vertebral fractures [27]. Similarly, López-Gómez et al. [28] reported distinct bone metabolic profiles: postmenopausal women with obesity presented reduced bone formation markers, whereas older obese women presented elevated bone resorption markers, suggesting a change in bone turnover dynamics that may contribute to fracture susceptibility. In addition, high BMI may be related to low muscle quality because of muscle fat infiltration. Low muscle quality is also related to a high risk of fracture [29]. Our data revealed a slightly high BMI in subjects with low RCs. The decreased fracture risk in subjects with high RCs may be partly due to a normal BMI.

How RCs affect the risk of fracture is unclear. Our data showed that the association between RC and fracture risk was mainly observed in female subjects. Estrogen plays an important role in bone metabolism. Cholesterol is a key precursor of estrogen [30]. Most of our population consists of elderly people. However, this does not mean that there is no estrogen in the body. The estrogen level decreases after menopause. Those subjects with a high RC may have a high level of estrogen. RC may decrease fracture risk by increasing estrogen levels. Estrogen not only regulates trabecular BMD but also affects cortical volumetric BMD and cortical porosity [31,32]. In addition, estrogen can also improve bone biomechanical strength [32]. All these factors play critical roles in fracture occurrence. Moreover, cholesterol also serves as the fundamental precursor for androgen synthesis [33]. Testosterone can promote the differentiation of osteoblasts and suppress osteoclast formation [34]. Bone microstructure or bone quality is another critical determinant of fracture. Bone remodeling depends on osteoblasts, marrow stromal cells and osteoclast formation and differentiation, which all require cholesterol. RC may decrease the fracture risk by directly affecting the bone microstructure [11].

We only observed an association between RC and fracture risk in female population. The possible reasons for sex-difference were unclear. One probability is the small sample size of fracture cases. Another reason may be the estrogen. Cholesterol is a key precursor of estrogen [30]. High RC may be related to high estrogen levels.

A recent expert consensus report for lung cancer screening in Asia has been published [35]. The screening strategies used differ across Asian countries. Age and pack-year smoking history are the main considerations. The expert consensus also revealed that nonsmokers aged 50--75 years with a family history of lung cancer should be included in screening programs. However, in China, CT lung cancer screening is recommended for individuals aged 50–80 years [36]. Subjects at those ages were also susceptible to osteoporotic fracture. An opportunistic assessment of fracture risk is helpful for the early identification of susceptible people.

### Advantages and limitations

This study has several advantages, such as its longitudinal design and large sample size. This study has several limitations. First, this study did not investigate the relationships between RC or BMD and osteoporosis because BMD data were not obtained. Second, this study reported only the association between RC and vertebral fracture risk. However, whether such associations exist between RC and nonspinal fractures, such as hip fracture or wrist fracture, has not been studied. Third, routine CT scans cannot identify fresh fractures, which may require surgery. We did not analyze the association between remnant cholesterol and vertebral fractures that required surgery. Fourth, although several variables were adjusted, other factors, such as dietary habits, smoking and drinking habits, physical exercise, abdominal obesity and body mass index, were not controlled. Our results showed that the association between RC and fracture risk was mainly observed in female subjects. In our country, smoking and drinking are not common in the female population. Smoking and drinking may not have played important roles in our study. Fifth, we assessed bone mass, but we did not assess primary or secondary osteoporosis. Sixth, the hyperlipidemic treatment status of patients at baseline and during follow-up was not assessed. Seventh, we only assessed fractures from T1--L2. Osteoporotic fractures most commonly occur in the vertebrae of the thoracic and lumbar spine, particularly in the lower thoracic spine (T10-T12) and upper lumbar spine (L1-L2) [37]. In addition, we did not consider menopausal status in the female population. Our population was aged 50 years or above. Most female populations at those ages have already reached menopause or are approaching menopause. Moreover, the underlying mechanisms were not studied. Finally, the sample size for the fracture population was relatively small, especially for the male population.

## Conclusion

In summary, this study revealed that subjects with high RCs had a lower risk of vertebral fracture than did those with low RCs. The RC level and the RC/cholesterol ratio were independently negatively associated with the risk of vertebral fracture. The associations between the RC or the RC/cholesterol ratio and fracture risk were mainly observed in female subjects. Monitoring RC levels may be useful for identifying subjects with high-risk fractures earlier, particularly women. However, further studies are needed to reveal the mechanisms by which remnant cholesterol affects bone metabolism and fracture risk.

## Supporting information

S1 File Raw data. (XLSX)
